# Distinctive characteristics of granulation tissue in laparotomy wounds with underlying oncological processes

**DOI:** 10.25122/jml-2022-0200

**Published:** 2023-02

**Authors:** Igor Kalynovych Morar, Oleksandr Ivanovich Ivashchuk, Yurii Yevhenovych Rohovyi, Volodymyr Yuriyovych Bodiaka, Aliona Andriivna Antoniv

**Affiliations:** 1 Department of Oncology and Radiology, Bukovinian State Medical University, Chernivtsi, Ukraine; 2 Department of Pathological Physiology, Bukovinian State Medical University, Chernivtsi, Ukraine; 3 Department of Internal Medicine, Clinical Pharmacology and Occupational Diseases, Bukovinian State Medical University, Chernivtsi, Ukraine

**Keywords:** granulation tissue, laparotomy wound, oncological process

## Abstract

This study aimed to investigate the effects of malignant neoplasms on the morphological characteristics of laparotomy wound granulation tissue in the muscular-aponeurotic layer. The study involved a sample of 34 deceased individuals who had undergone abdominal organ surgery. Biopsy samples were taken from the muscular-aponeurotic layer of the anterior abdominal wall and subjected to histological examination, including staining with hematoxylin and eosin and methylene blue/Chromotrope 2B using N.Z. Slinchenko’s method. Descriptive methods and morphometry were used to evaluate pathomorphological changes. The results suggest that malignant neoplasms significantly impede and decelerate the maturation of laparotomy wound granulation tissue. Surgeries performed at the late stages of abdominal organ malignant neoplasms result in an uneven and slow maturation of the tissue, characterized by a higher prevalence of lymphoid cells, increased blood vessel volume, reduced optical density of stained collagen fibers, and pronounced chromotropophilia of collagen fibers. These distinct features should be considered to prevent postoperative eventration, a complication that is more likely to occur in this patient group. Clinicians should be aware of the possible consequences of malignant neoplasms on laparotomy wound granulation tissue, which may require additional measures to prevent postoperative complications in these patients.

## INTRODUCTION

Postoperative eventration remains one of the most dangerous and threatening complications of abdominal surgery despite advancements in modern medicine. The mortality rate associated with postoperative eventration is estimated at 24%, and some authors suggest it to be as high as 65% [1-3]. General risk factors for postoperative eventration include old age, comorbidities, urgent surgery, relaparotomy, pneumonia, diabetes mellitus, cachexia, etc. [4-7].

Patients with oncological diseases of the abdominal organs are known to suffer from secondary immune deficiency, cachexia, anemia, etc., which is likely to influence the rate of regeneration and the risk of development of purulent-septic complications in the postoperative wound [8-11].

Understanding the morphology of granulation tissue in patients with malignant neoplasms of the abdominal organs may aid in the prevention of postoperative complications. Therefore, this study aimed to investigate the morphological features of the granulation tissue of the laparotomy wound in the muscular-aponeurotic layer after the removal of malignant neoplasms of the abdominal organs [12,13].

## MATERIAL AND METHODS

To achieve the research objective, we conducted a post-mortem examination of 34 deceased individuals who underwent abdominal organ surgery. The main group included 16 patients with malignant neoplasms of the abdominal organs at stages ІІІ-IV, who died within the first 7 days of the early postoperative period. The comparison group included 18 patients with acute non-oncological surgical pathology of the abdominal organs, who also died within the first 7 days of the early postoperative period [14,15]. Table 1 presents the distribution of patients according to the surgery performed on the abdominal organs. In this study, there were 19 (55,9%) females and 15 (44,1%) males. The average age of the individuals was 61.3 ± 2.08 years, and the patient groups were representative with respect to gender and age. All patients received standard postoperative treatment according to established protocols for patients with urgent surgical pathology of the abdominal organs during their hospitalization.

**Table 1:** Distribution of patients in both groups depending on the surgery performed, abs., %.

Surgery performed	Group of patients		abs.	%
	Comparison	Main		
**Distal stomach resection**	6	3	9	26.5
**Gastrectomy**	1	2	3	8.8
**Enterectomy**	3	1	4	11.8
**Right hemicolectomy (colonic resection)**	2	4	6	17.6
**Left hemicolectomy**	3	2	5	14.7
**Hartmann’s operation (rectosigmoid colon resection)**	3	4	7	20.6
**Total**	18	16	34	100

### Methods of morphological examination

The biopsy material obtained from the muscular-aponeurotic layer of the anterior abdominal wall for histological examination was fixed in a 10% neutral formalin solution. Paraffinic sections were prepared and stained with hematoxylin and eosin to visualize tissue structure. Additionally, N.Z. Slinchenko’s method of histological specimen staining with methylene blue/Chromotrope 2B was used to identify collagen fibers and fibrin. The morphological changes were analyzed using a descriptive method [16], and morphometry was performed using the computerized program ImageJ 1.48 v for computed microdensitometry to determine the optical density (OD) of collagen fibers. The specific volume of blood vessels in the granulation tissue (%) was determined using computed plane geometry, while the number of different types of granulation tissue cells was counted based on the surface area of the histological section (10000 µm^2^) [17].

### Statistical analysis

Statistical analysis was performed using Microsoft Excel and PAST software package. To assess normality, the Shapiro-Wilk test was conducted, and the Mann-Whitney test was utilized to compare differences between groups. A p-value of ≤ 0.05, which is generally accepted in medical-biological studies, was used as the threshold for determining the reliability of the results [18].

## RESULTS

**Figure 1:**
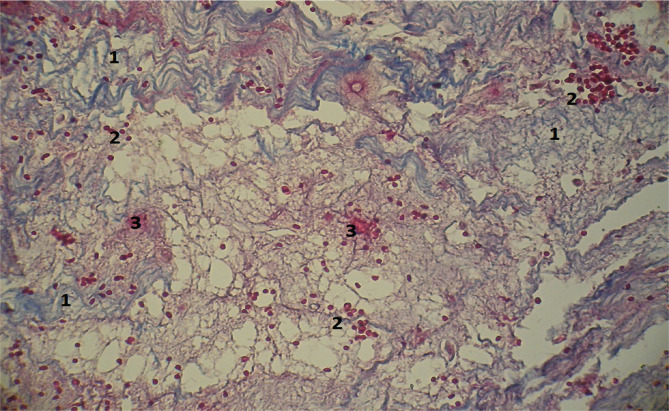
Microscopic view of the laparotomy wound granulation tissue on the first day of the early postoperative period. Methylene blue/Chromotrope 2B staining according to N.Z. Slinchenko’s method. The image shows abundant fibrin (1), a low number of lymphocytes (2), and areas of hemorrhage (3). Magnification: (Ob) 20×, (Oc) 10×.

**Figure 2:**
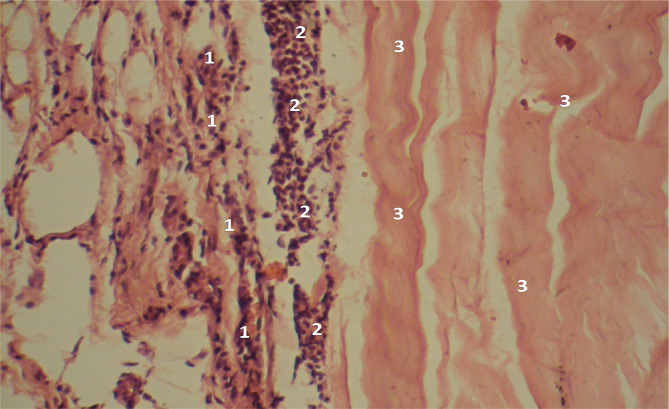
Microscopic view of laparotomy wound granulation tissue on the 3rd-4th day of the early postoperative period. Hematoxylin and eosin staining. The image shows an area of more mature granulation tissue (1) transitioning into an area of immature granulation tissue (2) in the muscular tissue layer (3). Magnification: (Ob) 20×, (Oc) 10×.

The microscopic pictures of the specimens obtained from deceased individuals in both groups during the first day of the early postoperative period showed comparable features, such as a high concentration of fibrin, minimal lymphocyte presence, and areas with hemorrhages (Figure 1). However, on the 2-3rd days, uneven maturation of the granulation tissue was observed in both groups, characterized by the presence of very immature foci (corresponding to 2-3 days – predominantly lymphoid cells) and moderately mature foci (corresponding to 5-7 days – predominantly fibroblasts) (Figure 2).

**Figure 3:**
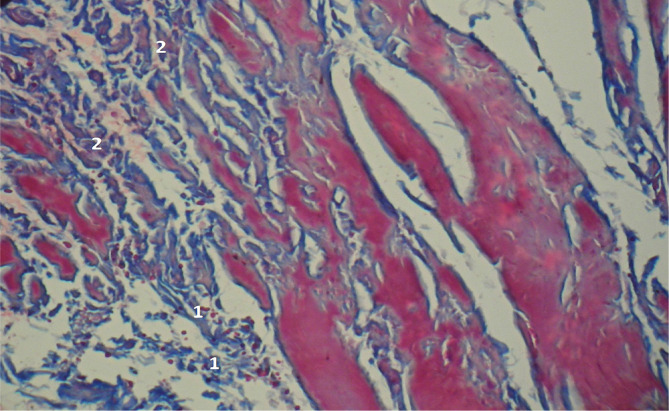
Microscopic view of the laparotomy wound granulation tissue on the 3-4th day of the early postoperative period. Methylene blue/Chromotrope 2B staining according to N.Z. Slinchenko’s method. The image shows the transition from young granulation tissue (1) to connective tissue (2) and marked chromotropophilia. Magnification: (Ob) 10×, (Oc) 10×.

**Figure 4:**
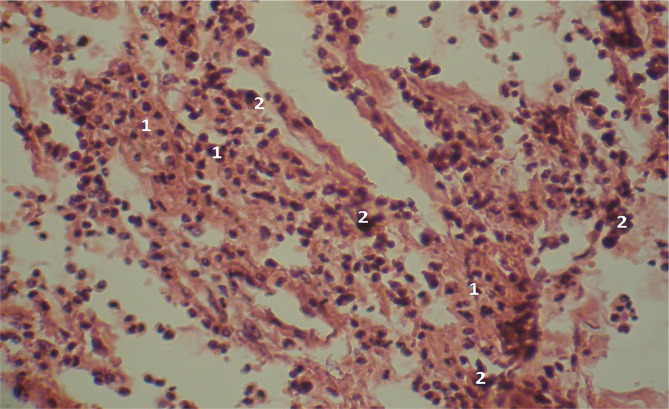
Microscopic view of the laparotomy wound granulation tissue on the 2-3rd day of the early postoperative period. Immature granulation tissue with numerous lymphoid cells (1) and a small number of fibroblasts (2) is visible. Hematoxylin and eosin staining. Magnification: (Ob) 20×, (Oc) 10×.

Methylene blue/Chromotrope 2B staining revealed elongated collagen fibers and an accumulation of fibroblasts, lymphocytes, and a number of lymphoid cells and macrophages, which is specific for the immature granulation tissue (Figure 3). The microscopic examination also revealed evenly immature granulation tissue with a significant number of lymphoid cells and macrophages, along with a limited presence of fibroblasts (as illustrated in Figure 4).

**Figure 5:**
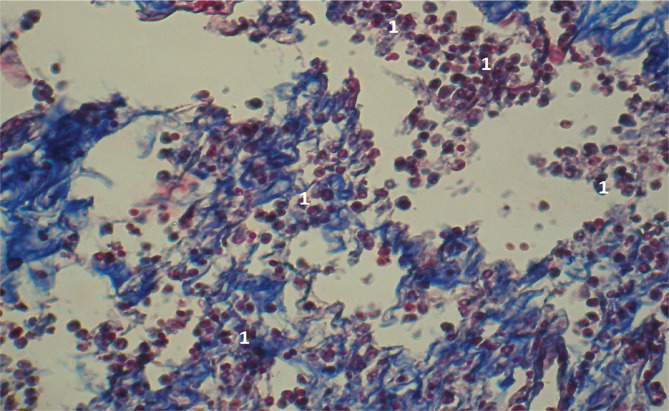
Microscopic view of the laparotomy wound granulation tissue on the 2-3rd day of the early postoperative period. Methylene blue/Chromotrope 2B staining according to N.Z. Slinchenko’s method. The image shows a high number of lymphoid cells (1) and absence of formed collagen fibers. Magnification: (Ob) 10×, (Oc) 10×.

The formation of collagen fibers was not well-defined, and a considerable number of lymphoid cells were observed using the Methylene blue/Chromotrope 2B-stained microscopic slides according to N.Z. Slinchenko's method (Figure 5).

**Figure 6:**
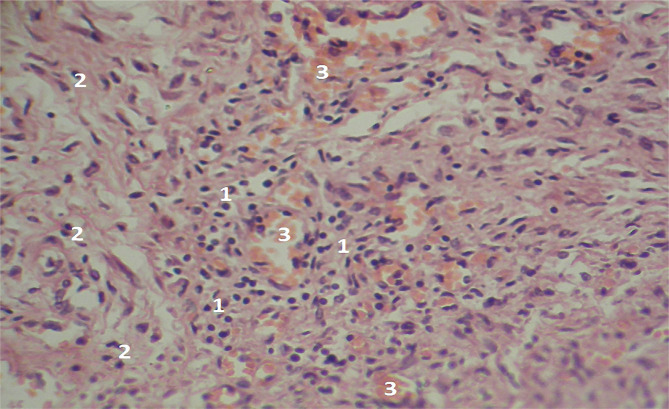
Microscopic view of the laparotomy wound granulation tissue on the 4-5th day of the early postoperative period. Hematoxylin and eosin staining shows uneven maturation of the young granulation tissue (1) with normal and elongated fibroblasts (2). Subtle muscle fibers are also visible (3). Magnification: (Ob) 20×, (Oc) 10×.

**Figure 7:**
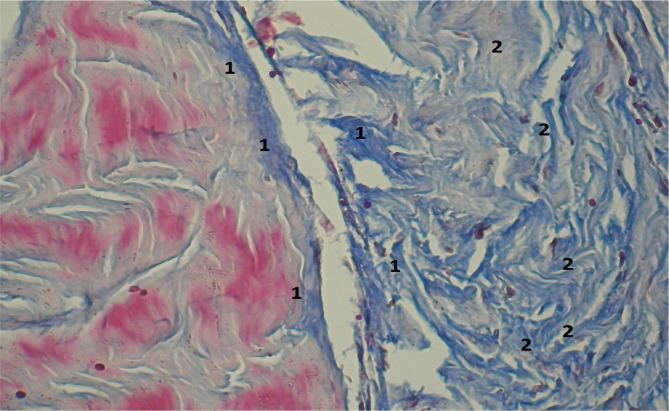
Microscopic view of the laparotomy wound granulation tissue on the 4-5th day of the early postoperative period. The image shows the area of fresh and mature granulation tissue with marked chromotropophilia. Methylene blue/Chromotrope 2B staining according to N.Z. Slinchenko’s method. Magnification: (Ob) 10×, (Oc) 10×.

**Figure 8:**
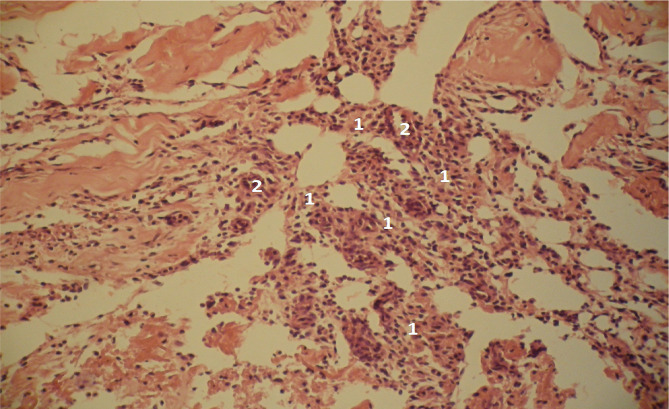
Microscopic view of the laparotomy wound granulation tissue on the 2-3rd day of the early postoperative period in physical bodies of deceased individuals. Hematoxylin and eosin staining. The lymphoid cells and fibroblasts (1) are evenly distributed, indicating regular maturation of the granulation tissue. Blood vessels (2) are also visible. Magnification: (Ob) 20×, (Oc) 10×.

During the 4-5th day of the early postoperative period, microscopic images in the main group displayed relatively more fibroblasts, fewer irregularly located lymphoid cells, and subtle muscle fibers (Figure 6). The slow maturation of blood vessels and marked chromotropophilia of collagen fibers were also observed (Figure 7). In the comparison group, there was a regular distribution of lymphoid cells and fibroblasts, indicating a more uniformly formed granulation tissue (Figure 8).

**Figure 9:**
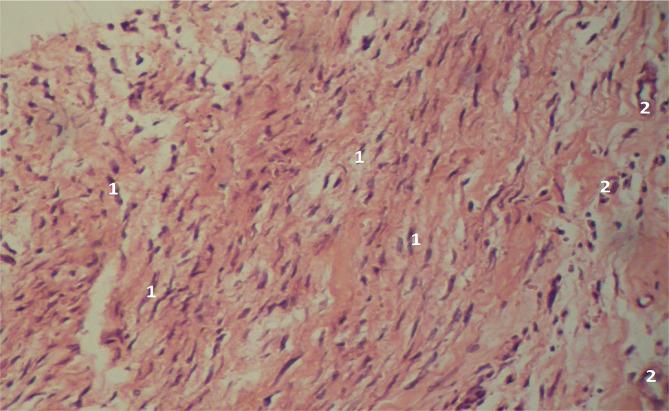
Microscopic view of the laparotomy wound granulation tissue on the 6-7th day of the early postoperative period in physical bodies of deceased individuals. Hematoxylin and eosin staining. The image shows a predominance of fibroblasts (1) and a low number of lymphoid cells, along with visible blood vessels (2). Magnification: (Ob) 20×, (Oc) 10×.

**Figure 10:**
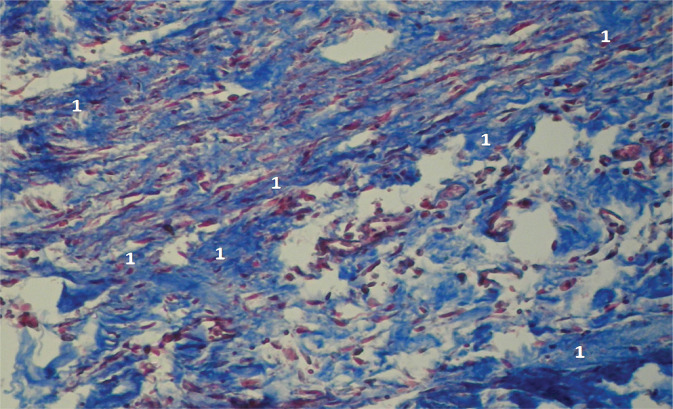
Microscopic view of laparotomy wound granulation tissue on days 6-7 of early postoperative period. The image shows intensively stained collagen fibers (1) and marked chromotropophilia, characteristic of later stages of tissue maturation. Stained with Methylene blue/Chromotrope 2B using N.Z. Slinchenko’s method. Magnification: (Ob) 10×, (Oc) 10×.

**Figure 11:**
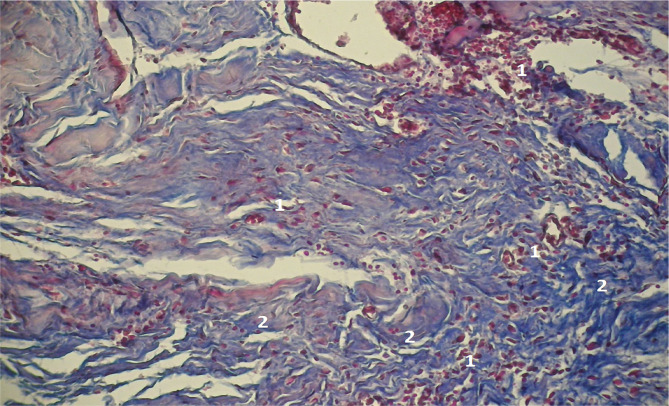
Microscopic view of the laparotomy wound granulation tissue on the 7th-8th day of the early postoperative period. Mature granulation tissue is visible, with fibroblasts (1) and collagen fibers (2) present and no chromotropophilia registered. Magnification: (Ob) 10×, (Oc) 10×.

During the later stages of the early postoperative period (6-7th days), the microscopic examination in the main group showed a less mature granulation tissue compared to the comparison group, as evidenced by the higher concentration of lymphoid cells and fibroblasts, and a clear chromotropophilia of collagen (Figure 9-11).

A morphometric examination was performed to obtain an objective comparison of the granulation tissue maturation processes in the muscular-aponeurotic layer of the laparotomy wound [19,20]. The findings are presented in Table 2. The microscopic examination of the main group revealed a higher number of cells during all observation periods, including lymphoid cells, plasmatic cells, macrophages, fibroblasts, and single polymorphonuclear leukocytes. The number of cells was significantly greater during the later stage of observation (4-7th days) in both groups, compared to the early stage (1-3rd days).

**Table 2:** Cell count per unit area of laparotomy wound granulation tissue in patients from both groups at different observation periods (M±m) x 10,000 mcm^2^.

Group of patients	Postoperative period	
	1-3^rd^ days	4-7^th^ days
**Comparison**	55.6±2.28	46.2±2.39 p1<0.05
**Main**	73.7±2.83 p<0.001	56.4±2.76 p<0.05; p1<0.01

n – number of observations; p – against the indices of comparison; p1 – against the indices of the 1-4th days of observation.

The results in Table 3 indicate a significantly lower optical density of the collagen fibers stained with Methylene blue in the microscopic slides from the main group. The optical density of staining of collagen fibers was higher during later observation periods, although this difference was statistically significant only in the comparison group.

**Table 3:** Optical density of the stained collagen fibers of the laparotomy wound granulation tissue in patients of both groups at different periods of observation (M±m), in the units of optical density.

Group of patients	Postoperative period	
	1-3^rd^ days	4-7^th^ days
**Comparison**	0.25±0.017	0.33±0.015 p1<0.05
**Main**	0.16±0.024 p<0.05	0.21±0.017 p<0.01; p1>0.05

n – number of observations; p – against the indices of comparison; p1 – against the indices of the 1-4th days of observation.

Further analysis of the study results, as presented in Table 4, revealed a significantly higher specific volume of the granulation tissue blood vessels in the microscopic slides. This increase was observed in both groups during the later observation periods.

**Table 4:** The specific volume of the granulation tissue blood vessels in both groups at different periods of observation, (M±m), %.

Group of patients	Postoperative period	
	1-3^rd^ days	4-7^th^ days
**Comparison**	8.8±0.71	11.9±0.32 p1<0.05
**Main**	11.8±0.78 p<0.05	14.8±0.85 p<0.05; p1<0.05

n – number of observations; p – against the indices of comparison; p1 – against the indices of the 1-4th days of observation.

## DISCUSSION

Despite advances in modern medicine, postoperative eventration remains one of the most dangerous and serious complications of abdominal surgery. General risk factors associated with the development of postoperative eventration include advanced age, comorbidities, urgent surgery, relaparotomy, pneumonia, diabetes mellitus, and cachexia, among others [21,22]. Surgery for abdominal wall eventrations is not considered as urgent in adult patients. The maturation of laparotomy wound granulation tissue is characterized by uneven and slow development, with a reliable prevalence of lymphoid-type cells, an increase in the specific volume of blood vessels, and a decrease in the optical density of stained collagen fibers.

Thus, summing up the results of the study it should be noted that malignant neoplasms considerably inhibit and slow down the processes of the laparotomy granulation tissue maturation. This is characterized by a reliable increase in the number of cells (especially of the lymphoid type), as well as an increase in the specific volume of blood vessels and a decrease in the optical density of the stained collagen fibers. These findings suggest that the vascular-exudative inflammation reaction is not effectively completed in patients with malignant neoplasms. In addition, these processes are accompanied by uneven maturation of granulation tissue, expressed by the chromotropophilia of collagen fibers [23-26].

Moreover, uneven maturation of the granulation tissue and marked chromotropophilia of the collagen fibers are registered in the microscopic slides of physical bodies of the deceased individuals who suffered from malignant neoplasms of the abdominal organs. When staining sectioned slides with water blue/chromotrope 2B, the microscopic analysis revealed the presence of elongated collagen fibers and accumulation of fibroblasts, lymphocytes, and a significant number of lymphoid cells and macrophages. These characteristics are indicative of the presence of immature granulation tissue, which can impede the process of wound healing.

Slower maturation of the laparotomy wound granulation tissue is explained by larger surgical trauma caused by a complete removal of a malignant neoplasm with underlying pathological changes in the body resulting in their considerable deterioration [27-30].

This distinctive characteristic should be carefully taken into consideration in order to prevent the development of postoperative eventration, which is significantly more likely to occur in this patient population.

## CONCLUSIONS

Our study found that surgical intervention for malignant neoplasms of the abdominal organs at advanced stages is associated with a slower and uneven maturation of the laparotomy wound granulation tissue. This was demonstrated by an increased number of cells, mainly lymphoid, an increase in the specific volume of blood vessels, and a decrease in the optical density of stained collagen fibers. Furthermore, marked chromotropophilia of the collagen fibers was observed. These findings indicate a higher risk of postoperative eventration in patients with malignant neoplasms of the abdominal organs at advanced stages compared to those without oncological pathology in the same location. Our study highlights the importance of careful monitoring and management of wound healing in this patient population to reduce the risk of postoperative complications.

## Data Availability

The data of this study is available by request.

## References

[1] Rivilla MB, Martinez-Barroso K, Morales AP, Gallego FJM (2018). Abdominal eventration with massive visceral content. Cir Esp (Engl Ed).

[2] Morar IK, Ivashchuk OI, Davydenko IS, Bodiaka VYu, Vlasov VV (2015). Specific characteristics of granulation tissue morphology round the elements of perforated graft after performing plastic surgery on the anterior abdominal wall against the ground of malignant tumoroces process. Clinical anatomy and operative surgery.

[3] Voitiv YaYu, Dyadyk OO (2020). Features of aponeurosis pathomorphological changes in patients with eventration. Clinical and experimental pathology.

[4] Vorovsky AA, Shaprinsky VA, Yatskov DA (2017). Surgical treatment of eventrations and eviscerations in purulent-inflammatory diseases of the abdominal wall and abdominal cavity. Kharkiv Surgical School.

[5] Sabra H, Alimoradi M, El-Helou E, Azaki R (2020). Perforated sigmoid colon cancer presenting as an incarcerated inguinal hernia: A case report. Int J Surg Case Rep.

[6] Shaprynskiy VO, Vorovskiy OO, Pashynskyi YaM (2019). Intraperitoneal hypertension as the reason of eventration development in elderly patients. Clinical anatomy and operative surgery.

[7] Mericli AF, Baumann DP, Butler CE (2018). Reconstruction of the Abdominal Wall after Oncologic Resection: Defect Classification and Management Strategies. Plast Reconstr Surg.

[8] Bourra K, El Mazouz S (2017). Latissimus dorsi flap in reconstruction following treatment of giant tumor of the abdominal wall: about a rare case. Pan Afr Med J.

[9] Roubaud MS, Baumann DP (2018). Flap Reconstruction of the Abdominal Wall. Semin Plast Surg.

[10] Yang Z, Wang F, Liu S, Guan W (2019). Comparative clinical features and short-term outcomes of gastric and small intestinal gastrointestinal stromal tumours: a retrospective study. Scientific Reports.

[11] Gál P, Varinská L, Fáber L, Novak S (2017). How Signaling Molecules Regulate Tumor Microenvironment: Parallels to Wound Repair. Molecules.

[12] Song D, Li Z, Zhou X, Zhang Y (2019). Application of pedicled anterolateral thigh myocutaneous flap for full-thickness abdominal wall reconstruction after tumor resection. Zhongguo Xiu Fu Chong Jian Wai Ke Za Zhi.

[13] Goto A, Matsuhashi N, Takahashi T, Tanahashi T (2020). Feasibility of the Reconstruction with Fascia Lata Patch on the Abdominal Wall Defect After Resection of the Abdominal Desmoid Tumor. Clin Exp Gastroenterol.

[14] Morar I, Ivashchuk O, Bodiaka Yu, Antoniv A, Chuprovska Y (2022). The role of oncological process in occurrence of postoperative eventration. Georgian Med News.

[15] Malishevsky IA (2020). Clinical and epidemiological analysis of malignant neoplasms of the abdominal organs. Clinical and experimental pathology.

[16] Vlasov VV, Morar IK, Davydenko IS, Bodyaka VY, Pokhodun KA (2019). Granulation tissue morphology of the laparotomic wound with different types of sutures. Surgery of Ukraine.

[17] Zhang Q, Zheng F, Motulsky EH, Gregori G (2018). A Novel Strategy for Quantifying Choriocapillaris Flow Voids Using Swept-Source OCT Angiography. Invest Ophthalmol Vis Sci.

[18] Sofalvi S, Schueler HE (2021). Assessment of Bioanalytical Method Validation Data Utilizing Heteroscedastic Seven-Point Linear Calibration Curves by EZSTATSG1 Customized Microsoft Excel Template. J Anal Toxicol.

[19] Martis G, Laczik R, Damjanovich L (2017). Significance of the computed tomography assisted morphometry in the surgical planning of eventrated abdominal wall hernias. Orvosi Hetilap.

[20] Khomenko IP, Tsema IeV, Shapovalov VYu, Tertyshyn SV (2019). Usage of full-thickness Keystone flap procedure in anterior abdominal wall reconstruction (case report). Surgery of Ukraine.

[21] Cui H, Zhang KC, Cao B, Deng H (2021). Risk factors of postoperative complication after total gastrectomy in advanced gastric cancer patients receiving neoadjuvant chemotherapy. Zhonghua Wei Chang Wai Ke Za Zhi.

[22] Kang SC, Kim HI, Kim MG (2016). Low Serum Albumin Level, Male Sex, and Total Gastrectomy Are Risk Factors of Severe Postoperative Complications in Elderly Gastric Cancer Patients. J Gastric Cancer.

[23] Khansa I, Janis JE (2015). Modern reconstructive techniques for abdominal wall defects after oncologic resection. J Surg Oncol.

[24] Kalemci S, Ergun KE, Kizilay F, Yildiz B, Simsir A (2022). Analysis of risk factors of abdominal wound dehiscence after radical cystectomy. Rev Assoc Med Bras 1992.

[25] Boyko VV, Ivanova YuV, Tymchtnko ME (2019). Surgical treatment of high-risk patients in patients. Kharkiv Surgical School.

[26] O'Connell S, Islam S, Sewell B, Farr A (2022). Hughes abdominal closure versus standard mass closure to reduce incisional hernias following surgery for colorectal cancer: the HART RCT. Health Technol Assess.

[27] Colozzi S, Clementi M, Cianca G, De Santis G (2016). Early Postoperative Eventration: Surgical Treatment with Use of Biological Prosthesis. J Clin Case Rep.

[28] Tabola R, Augoff K, Lewandowski A, Ziolkowski P (2016). Esophageal anastomosis-how the granulation phase of wound healing improves the incidence of anastomotic leakage. Oncology Letters.

[29] Gray M, Marland JRK, Murray AF, Argyle DJ, Potter MA (2021). Predictive and Diagnostic Biomarkers of Anastomotic Leakage: A Precision Medicine Approach for Colorectal Cancer Patients. J Pers Med.

[30] Smith SR, Pockney P, Holmes R, Doig F (2018). Biomarkers and anastomotic leakage in colorectal surgery: C-reactive protein trajectory is the gold standard. ANZ J Surg.

